# Estrogens and Progestogens in Triple Negative Breast Cancer: Do They Harm?

**DOI:** 10.3390/cancers13112506

**Published:** 2021-05-21

**Authors:** Mark van Barele, Bernadette A. M. Heemskerk-Gerritsen, Yvonne V. Louwers, Mijntje B. Vastbinder, John W. M. Martens, Maartje J. Hooning, Agnes Jager

**Affiliations:** 1 Department of Medical Oncology, Erasmus MC Cancer Institute, University Medical Centre, Dr. Molewaterplein 40, 3015 GD Rotterdam, The Netherlands; m.vanbarele@erasmusmc.nl (M.v.B.); b.heemskerk-gerritsen@erasmusmc.nl (B.A.M.H.-G.); j.martens@erasmusmc.nl (J.W.M.M.); m.hooning@erasmusmc.nl (M.J.H.); 2 Department of Obstetrics and Gynaecology, Erasmus MC, University Medical Centre, Dr. Molewaterplein 40, 3015 GD Rotterdam, The Netherlands; y.louwers@erasmusmc.nl; 3 Department of Internal Medicine, Ijsselland Hospital, Prins Constantijnweg 2, 2906 ZC Capelle aan den IJssel, The Netherlands; mvastbinder@ysl.nl

**Keywords:** triple-negative, hormone-receptor negative breast cancer, ERβ, RANK/RANKL, GPER, AR, estrogens, progestogens

## Abstract

**Simple Summary:**

Young women treated for breast cancer may experience early menopause, which can negatively impact quality of life. The usual treatment for early menopause is hormone replacement. For hormone-sensitive breast cancers, however, hormones are not given as they can adversely affect the prognosis. In the case of triple-negative breast cancers (TNBC), which do not express the estrogen receptor (ER) and progesterone receptor (PR), research has not been able to show whether hormone replacement is safe yet. Theoretically, however, there are many possible mechanisms by which female hormones can have an effect on TNBC. We therefore reviewed the clinical and preclinical data investigating possible (in)direct effects of estrogens and progestogens on the course of TNBC. The ultimate aim is to provide practical recommendation on how best to treat chemotherapy-induced early menopause in TNBC patients.

**Abstract:**

Triple-negative breast cancers (TNBC) occur more frequently in younger women and do not express estrogen receptor (ER) nor progesterone receptor (PR), and are therefore often considered hormone-insensitive. Treatment of premenopausal TNBC patients almost always includes chemotherapy, which may lead to premature ovarian insufficiency (POI) and can severely impact quality of life. Hormone replacement therapy (HRT) is contraindicated for patients with a history of hormone-sensitive breast cancer, but the data on safety for TNBC patients is inconclusive, with a few randomized trials showing increased risk-ratios with wide confidence intervals for recurrence after HRT. Here, we review the literature on alternative pathways from the classical ER/PR. We find that for both estrogens and progestogens, potential alternatives exist for exerting their effects on TNBC, ranging from receptor conversion, to alternative receptors capable of binding estrogens, as well as paracrine pathways, such as RANK/RANKL, which can cause progestogens to indirectly stimulate growth and metastasis of TNBC. Finally, HRT may also influence other hormones, such as androgens, and their effects on TNBCs expressing androgen receptors (AR). Concluding, the assumption that TNBC is completely hormone-insensitive is incorrect. However, the direction of the effects of the alternative pathways is not always clear, and will need to be investigated further.

## 1. Introduction

Triple-negative breast cancer (TNBC) comprises a subgroup of breast cancers (BC) identified by a lack of expression of the estrogen receptor (ER) and progesterone receptors (PR), and without amplification of Human Epidermal Growth Factor Receptor-2 (*HER2* or *ERBB2*). This subgroup of BC accounts for about 15–20% of BC cases, is diagnosed more often in young premenopausal women, and due to its aggressive nature, often requires (neo)adjuvant chemotherapy to improve long-term prognosis [1]. Lacking hormone receptors, these TNBCs are not susceptible to endocrine therapy [2]. Under the assumption that estrogens and progestogens have no growth stimulating potential on residual cancer cells, (recovery of) ovarian function (ovarian function within the context of this review refers to ovarian hormone steroidogenesis) in young TNBC patients is not actively suppressed. When no recovery of ovarian function occurs, HRT for menopausal complaints is occasionally considered, but no conclusive evidence supporting the safety really exists [3].

It would be of great clinical value to know whether estrogens and progestogens actually are harmless when it comes to recurrence risk in TNBC patients. While generally considered detrimental for ER-positive BC patients based on prospective studies [4,5,6], hormonal replacement therapy (HRT) could still be an option for HR-negative BC/TNBC patients, many of whom are young and at high risk of premature ovarian insufficiency (POI) due to toxicity of chemotherapy [7]. The excess morbidity, and perhaps even mortality [8], associated with iatrogenic/early menopause warrants a careful re-examination of the feasibility of HRT for these patients.

Our goal here is to review the scientific evidence from both clinical and preclinical studies regarding the effects of estrogens and progestogens in hormone receptor-negative BC and to arrive at a practical recommendation concerning maintaining a postmenopausal status or use of HRT in TNBC patients.

## 2. Clinical Evidence for a Role of Estrogen and Progesterone in TNBC

### 2.1. The Level of ER Expression in Primary TNBC

TNBC is logically considered to be hormone-receptor negative. However, there are actually two commonly used cut-off points for what is considered to be negative. The American Society of Clinical Oncology (ASCO) recommends only classifying tumors with an ER and/or PR expression less than 1% as negative, while in several European countries a cut-off of less than 10% ER and/or PR expression is used.

An important consequence to consider is that, theoretically, a TNBC expressing ER in up to 9% of cells could leave ER positive residual disease after (initial) treatment, suggesting that these patients might benefit from adjuvant endocrine therapy, and likewise high estrogen levels could have adverse effects. Indeed, a large meta-analysis of individual patient data showed that while tamoxifen’s beneficial effect was much weaker in what they defined as “ER-poor” (<10 femtomole ER per mg cytosol protein in ligand-binding assay) than in ER-positive disease, the effect was not completely absent [2]. It should be noted that protein expression by ligand-binding assay is correlated, but not directly translatable to a percentage ER expression.

However, several studies showed that HER2-negative tumors with weak ER expression (1–9% of cells) behave mainly like pure TNBC in its response to endocrine therapy and chemotherapy [9,10], although they can be genetically heterogenous [11]. The variability in cut-off value for ER/PR-positivity therefore most likely does not fully explain unexpected hormonal effects in ER-negative/TNBC patients.

### 2.2. ER Conversion of Primary TNBC

ER conversion, or receptor conversion in general, is the clinical phenomenon where metastatic lesions exhibit a different expression pattern of ER, PR, and/or HER2 than the primary BC. A meta-analysis on receptor expression between primary and metastatic lesions found that about 20% of the ER-negative primary tumors, showed ER expression on a metastatic lesion [12]. Whether this reflects a primarily estrogen-driven selection on the initial presence of ER-positive cells within the TNBC, or whether this is the result of a “true” receptor conversion (i.e., resulting from epigenetic upregulation, although selection can/will then still occur) is unclear, and could very well be the result of a combination of both. Although it may seem plausible that a 1–9% ER-expressing TNBC may have a higher risk of ‘conversion’, no significant difference was found based on either a 1% or 10% threshold for positivity [12].

Therefore, receptor conversion could have important consequences, as ovarian recovery would have been harmful in case ER-positive residual disease emerges from what was believed to be a hormone-insensitive TNBC.

### 2.3. Effect of Recovery of Ovarian Function (Endogenous Hormone Exposure) after Chemotherapy

Studies investigating the effect of endogenous hormone exposure in hormone-receptor negative BC patients are scarce. They have mainly been performed in premenopausal patients, investigating persistence or recovery of the menstrual cycle after chemotherapy and its effect on prognosis. Most of the available data are from retrospective cohort studies. A meta-analysis done by Zhou et al. in 2015 included an ER-negative/PR-negative subgroup analysis of three studies, finding that recovery of ovarian function after chemotherapy did not appear to affect prognosis (pooled HR 0.97, 95% CI: 0.66–1.41) [13]. A more recent study did not find an effect of treatment-related amenorrhea on disease-free survival (DFS) or overall survival (OS) in their HR-negative subgroup analysis either (HR 0.92; 95% CI 0.70–1.20 for DFS, and HR 1.03; 95% CI 0.68–1.56 for OS) [14]. However, patients in these studies were classified as non-exposed to endogenous hormones when they experienced amenorrhea for a predefined period of time, usually being six months without a menstrual cycle, measured at twelve months after diagnosis/treatment. Patients with recurrence of menstrual cycles within this timeframe of six months were classified as exposed. This classification, however, does not take into account the recovery of the menstrual cycle after more than six months, which will also lead to endogenous hormone exposure and might impact prognosis. Considering this, the reference groups (i.e., non-exposed) in the above described studies then consist of patients with permanent amenorrhea as well as patients with temporary amenorrhea, with the latter likely exposed to hormones. This mixing of exposures may have increased the recurrence risk in the reference group, and will cause risk ratios to be biased towards the null (i.e., hazard ratio closer to 1). Although the results of these studies appear consistent, one might argue that misclassification bias caused an underestimation of any potential effects.

In light of this potential misclassification bias, it is of interest that two retrospective studies that considered temporary and permanent amenorrhea as separate exposures, found a trend towards an increased recurrence risk in women with HR-negative BC and a persistent menstrual cycle or recovery after amenorrhea (HR 1.45; 95% CI 0.83–2.54 and HR 1.73, 95% CI 0.86–3.48, respectively) [15,16]. Interestingly, a retrospective study on risk-reducing salpingo-oophorectomy (RRSO, i.e., preventive surgical ovarian and fallopian tube removal) after BC diagnosis in *BRCA*-mutation carriers found a strong protective effect on recurrence for ER-negative BC patients, but not ER-positive BC patients [17]. However, selection bias cannot be excluded in this study, and it can be argued that this bias would be stronger in ER-negative BC patients, as relapse in TNBC almost always occurs within the first five years after diagnosis [18], while mean time to RRSO was 6.1 years. This implies that patients with an inherently good prognosis survive until they can undergo RRSO, rather than RRSO causing them to survive. Furthermore, a meta-analysis of ovarian ablation found no beneficial effect for ER-negative BC patients treated with chemotherapy. A comparison with a non-chemotherapy group was not possible due to lack of data [19]. Essentially, the natural recovery of the menstrual cycle may have adverse effects in TNBC patients, as further demonstrated by the potential positive, although possibly biased effects of ovarian removal. The effects of hormone replacement in clinical studies may provide further insight.

### 2.4. Effect of Exogenous Hormone Exposure

For ER-positive BC, randomized controlled trials have demonstrated an increased risk of BC recurrence with HRT use after successful primary treatment, leading to the premature termination of the trials [4,5]. Combined with the abundance of evidence for the efficacy of adjuvant anti-hormonal therapy in ER-positive BC, this has eventually resulted in HRT being contraindicated for generally all women with a history of breast cancer. However, data on the potential harmful effect of HRT on BC prognosis among ER-negative BC are scarce, and showed inconclusive results. Kenemans et al. showed no significant negative effect of HRT in the subgroup analysis of ER-negative BC patients (RR 1.15; 95% CI 0.73–1.80) whereas Holmberg and colleagues showed a non-significant increased recurrence risk of 1.9 (95% CI 0.4–9.6) for HR-negative BC patients [4,5]. Furthermore, several studies have shown that young age (usually <40 years) may be an independent risk factor for recurrence in TNBC, even after adjusting for other risk factors, such as stage, grade (and family history) [20,21,22]. A more aggressive tumor biology among patients developing BC at a younger age has been suggested to be a plausible explanation [23]. However, significant differences in age-related factors such as premenopausal status may also play a role and should not be disregarded. A randomized clinical trial of hormone-receptor negative BC patients has in fact already demonstrated a positive effect of inducing temporary ovarian insufficiency using goserelin (a gonadotropin-releasing hormone analogue that inhibits ovarian hormone production) on prognosis [24]. Based on these clinical studies, insufficient evidence to justify prevention of spontaneous menstrual cycle recovery exists, while there is simultaneously too much uncertainty surrounding HRT for climacteric complaints in TNBC patients.

## 3. Preclinical Evidence for a Role of Estrogen and Progesterone in TNBC

### 3.1. Estrogen Receptor Beta in TNBC

**Figure 1:**
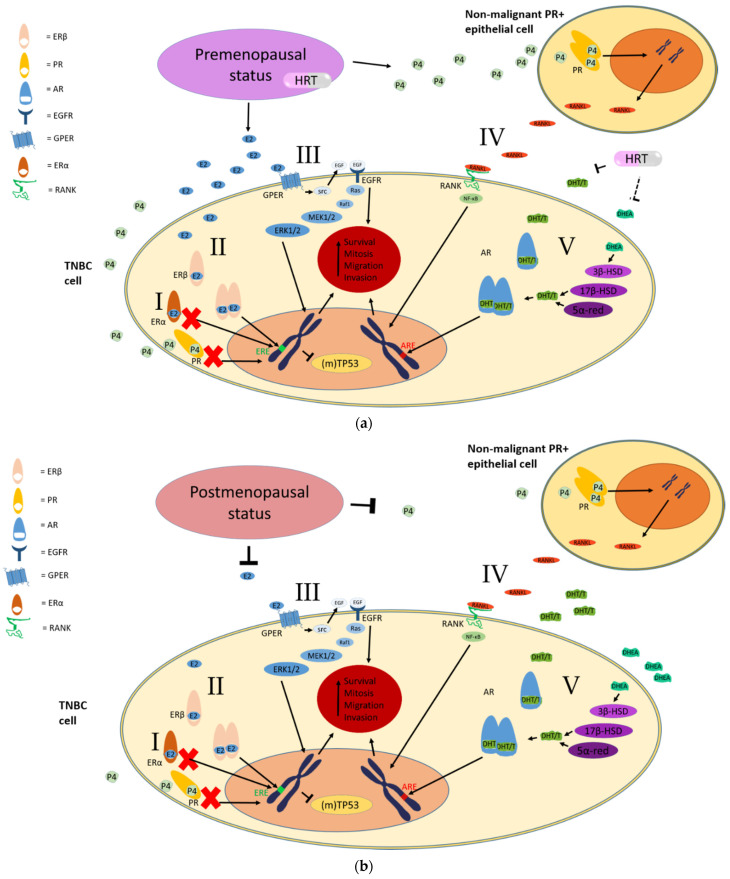
Schematic overview of the non-present canonical ER/PR pathways (indicated by the red crosses) and potential alternative pathways for estrogen and progestogen action in TNBC. Normal pointed arrows indicate stimulation, stunted arrows indicate inhibition (dashed arrow = weak or uncertain effect). Red crosses indicate the absence of the traditional hormonal pathway in TNBC. Inhibitory effect on (mutant)TP53 is most likely ERβ specific. (**a**) Stimulation of hormonal pathways in a physiological premenopausal state. (**b**) Inhibition of hormonal pathways in a physiological postmenopausal state. Abbreviations: HRT, hormone replacement therapy; ER, estrogen receptor; PR, progesterone receptor; AR, androgen receptor; E2, 17β-estradiol; P4, progesterone; DHT/T, dihydrotestosterone and/or testosterone; DHEA, dehydroepiandrosterone; ERE, estrogen response element; ARE, androgen response element; TNBC, triple-negative breast cancer; RANK, Receptor Activator of Nuclear Factor Kappa-B; RANKL, RANK-ligand; NF-κB, nuclear factor kappa-B; GPER, G protein-coupled estrogen receptor; EGFR, epithelial growth factor receptor; Src, SRC proto-oncogene, non-receptor tyrosine kinase; EGF, epithelial growth factor; Ras, Ras subfamily of small GTPases; Raf1, Raf-1 proto-oncogene, serine/threonine kinase; MEK1/2, mitogen-activated protein kinase kinase (MAPKK) 1, 2; ERK1/2, mitogen-activated protein kinase (MAPK) 1, 2; 3β-HSD, 3β-hydroxysteroid dehydrogenase; 17β-HSD, 17β-hydroxysteroid dehydrogenase; 5α-red, 5α-reductase; (m)TP53, (mutant)tumor protein 53.

Two distinct nuclear ER proteins are currently known to exist, the ERα protein (encoded by the *ESR1* gene), and the ERβ protein (encoded by the *ESR2* gene) (see Figure 1 for a schematic representation of both receptors, pathways I and II). Both receptors are activated by estrogens, including 17β-estradiol (E2). When the diagnosis of breast cancer is made, the status of the ERα protein of the tumor is determined using immunohistochemistry (IHC). ERα expression is associated with tumor differentiation and is a good predictor for tamoxifen response [25,26]. Tumors that do not express ERα can still express the ERβ protein (*ESR2*) [27], which is structurally and functionally different from ERα [28].

Up to 65% of ERα-negative/TNBC tumors may express some form of ERβ [27,29,30], although this may possibly be an overestimation due to non-specific antibody staining [31]. ERβ has multiple known isoforms (e.g., ERβ1, ERβ2, ERβ5), of which the exact function is not well-established yet. Only ERβ1 appears to have ligand-binding capability [29]. This ERβ1 isoform shows moderate or high nuclear expression in at least 20–25% of TNBC cases [32,33]. The other isoforms are capable of forming heterodimers with ERβ1 (and ERα), modulating its activity [34], and may act as prognostic factors [35]. It should be noted that splice variants/isoforms of ERα also exist. However, these are also detected by the antibody used for detection of ERα and, therefore, they are not present nor relevant in TNBC [36].

In the preclinical setting, the significance of ERβ expression in TNBC is not entirely clear. Many studies show a potential beneficial effect of ERβ expression on prognosis, through inhibition of the epithelial-to-mesenchymal-transition [37], through inhibition of invasion and migration [38,39] or through lowering proliferation rates [32,40,41]. Other, more specific benefits include inhibition of mutant p53 [42], blockade of the cell cycle [43] and/or activation of cystatins that block the TGFβ pathway [44,45]. The interaction of ERβ with p53 is interesting, as it would appear ERβ interferes with p53 function [46]. As *TP53* mutations are present in up to 80% of clinical TNBC samples [47], and present in frequently used TNBC cell lines [48,49], a beneficial effect of ERβ is to be expected. However, when wild-type p53 fulfils its role as a tumor-suppressor, this interference may result in tumor-promoting effects. Indeed, opposite effects of ERβ have also been reported, with ERβ1 activation stimulating TNBC proliferation [50,51], promoting TNBC cell survival [52] and increasing tumor aggressiveness [50,53]. In summary, the overall effect of ERβ on prognosis of TNBC in preclinical models appears inconclusive. Another possible explanation for these conflicting results is that the ERβ isoforms have differential expression patterns and effects on prognosis. A recent study reported both normal and high mRNA expression of ERβ2 and ERβ5 in several commonly used TNBC cell lines, but only very low (or no) ERβ1 expression [54]. Simultaneous downregulation of ERβ2 and ERβ5 resulted in reduced proliferation, migration and invasiveness of TNBC cell lines, while independent overexpression of both ERβ2 and ERβ5 caused the opposite effect. In contrast, overexpression of ERβ1 (using a viral vector) led to suppression of proliferation, migration and invasiveness of TNBC cell lines [54]. Analysis of a clinical database revealed similar patterns of ERβ isoform expression, although only ERβ2 appeared to be associated with a worse overall survival in TNBC patients [54]. Possible methodological causes for the inconsistent results of ERβ studies, such as non-specific antibodies, have been addressed elsewhere [31,33,55]. It should be noted that these issues may have affected results from the clinical studies discussed in the next paragraph as well.

Clinical studies of ERβ expression in TNBC patients also showed somewhat inconclusive results [27,50,56,57,58,59,60,61], but there appears to be more reliable evidence for an association with a worse prognosis [27,50,58,60,61]. Interestingly, there is evidence that this unfavorable prognosis might be favorably influenced by tamoxifen treatment. Although randomized studies are lacking, in all studies in which ERα-negative breast cancer patients were treated with tamoxifen, the disease-free and overall survival was better in patients with high levels of retrospectively measured ERβ1 or ERβ-total expression compared to those with a low level of ERβ [61,62,63,64]. Taken together, the clinical data appear to indicate an overall unfavorable effect on prognosis of ERβ-expression in TNBC tumors, which may be ameliorated by tamoxifen treatment. As we are currently lacking effective targeted therapies, new treatments for TNBC patients are most welcome. The main challenge will be confirming that it is indeed isoforms ERβ2 or ERβ5 that are detrimental to the patient, and whether ERβ1 is actually beneficial. Then, we will need to find ways to specifically inhibit ERβ2 or ERβ5, but not interfere with ERβ1′s possible anti-tumor effects. This specificity is important, as a clinical phase II trial of E2 treatment of metastatic TNBC patients failed to show a meaningful effect in an unselected, but mostly ERβ-positive population [65].

### 3.2. The G Protein-Coupled Estrogen Receptor (GPER1/GPR30)

Estrogen receptors, such as ERα belong to a family of nuclear receptors that act directly on the genome when activated, a process that is relatively slow. However, the observation of rapid effects of estrogen suggested a mode of action that is not directly on the level of the genome and the presence of a cell membrane-bound E2-receptor was theorized [66,67]. This receptor was later identified to be the G-protein-coupled receptor homolog, GPR30, now (also) named G-protein-coupled estrogen receptor (GPER) [68]. Although GPER is considered to cause mostly non-genomic (i.e., rapid signaling) effects upon E2 stimulation, non-genomic and genomic actions can overlap (ERα may also initiate rapid signaling) and rapid signaling eventually also leads to changes in transcriptional activity [69]. Discerning non-genomic and genomic effects in the results described here may therefore prove difficult, if not impossible, in for example results from animal studies. In the following discussion, the distinction between genomic and non-genomic actions of GPER activation is therefore not made. Rapid non-genomic signaling pathways (e.g., increasing cAMP levels, calcium mobilization) of GPER activation are reviewed in [69]. A variety of ligands, such as estrogens, can bind to this GPER. Stimulation of GPER activates a cascade of proteins (such as epidermal growth factor receptor (EGFR), see Figure 1, pathway III) that are associated with proliferation and survival [70], possibly with angiogenesis [71] as well as migration [72] and metastasis [73].

Interestingly, (17β-)estradiol (E2) has indeed been shown to promote growth of (basal-like) GPER-expressing TNBC cells in vitro [74,75]. Furthermore, knockdown of GPER using siRNA completely blocks E2-induced proliferation and prevented activation (phosphorylation) of EGFR in TNBC cell lines [76]. Up to 70% of TNBC has been reported to express GPER [74,77,78]. E2 could have a direct growth-stimulating effect in these TNBCs. However, GPER may be inhibited by ERβ expression [79], in which case the overall effect in the total TNBC population is diminished, as ERβ is expressed in up to 60% of TNBCs. In other words, E2 could directly stimulate growth via the GPER in TNBC, but possibly only when these tumors do not also express ERβ. The clinical implication is that GPER expression may worsen the prognosis of TNBC patients. Indeed, this appears to be the case, although reports of the effect of GPER expression on outcome in TNBC are scarce [80].

### 3.3. The Effect of the RANK/RANKL Pathway

A cell that is completely incapable of interacting with a certain hormone, can still be affected by it through receptive (healthy) neighboring cells in a paracrine manner. An important pathway capable of filling this role is that of Receptor Activator of Nuclear factor Kappa(κ)B (RANK) and its ligand, RANKL.

Normal differentiated mammary epithelial cells often express ER/PR. Progesterone binding to PR on these cells stimulates the production of RANKL [81,82]. RANKL in turn can bind to RANK on neighboring ER/PR-negative tumor cells and stimulate proliferation of these cells in a paracrine fashion (as demonstrated in Figure 1, pathway IV), reviewed in [83,84]. In addition to stimulating proliferation [82,85,86], the RANK/RANKL pathway also appears capable of protecting cancer cells from apoptosis due to chemotherapy or radiation [85]. Additionally, the RANK/RANKL pathway may induce stem-cell-like properties as well as the epithelial-mesenchymal-transition (EMT) that is associated with developing metastases [87,88].

RANK/RANKL has been positively associated with ER/PR-negative BC in clinical samples, both in terms of mRNA expression levels (which were about threefold higher than in ER/PR-positive BC) and presence of RANK protein by IHC (present about three times more often (60–72%) than in ER+/PR+ BC [88,89,90,91]). More specifically, between TNBC and non-TNBC, differences in mean mRNA expression of RANK by as much as a factor ten have been reported [89]. Furthermore, these authors and others have shown that RANK/RANKL expression is associated with decreased survival [85,88,89,90,92,93].

It has been shown that knockout of the progesterone receptor is associated with downregulation of RANKL expression [94]. Interestingly, in a randomized clinical trial with ER/PR-negative BC patients, those treated with chemotherapy plus gonadotropin-releasing hormone agonist/analogue (GnRH-a, inhibits ovarian production of estrogens and progesterone) had a better overall survival than those treated with chemotherapy without GnRH-a [24]. This seems counterintuitive at first, as recovery of menstruation occurred more often in the GnRH-a treated patients which increases hormonal (progestogen) exposure. However, the initial suppression of ovarian function and thereby suppressing progesterone levels may prevent upregulation of the RANK/RANKL pathway, rendering tumor cells of especially the basal type more vulnerable to undergoing apoptosis after being exposed to chemotherapy. GnRH-a may also have a direct toxic effect on TNBC, as many TNBCs appear to express GnRH receptors. However, both clinical and preclinical research regarding the effects of GnRH-a on GnRH-receptor positive TNBC is severely limited [95,96,97,98,99]. Nonetheless, it would be of great interest to repeat a study of GnRH-a among TNBC patients, with recurrence free survival as the primary endpoint, instead of recovery of ovarian function.

Interestingly, the RANK/RANKL pathway has also been implicated as an important vector of carcinogenesis in *BRCA1* mutation carriers. It was discovered that *BRCA1* mutation carriers harbor a subset of mammary progenitor cells that are the likely origin for basal-like BCs in these women [100,101]. More recently, it became apparent that it is in fact the RANK-positive subset among these progenitor cells, which have cancer-like properties, and are therefore the most likely origin of *BRCA1*-related BC [102]. Even more interesting was the observation that proliferation rates in biopsy samples from *BRCA1* mutation carriers were reduced when they were treated with an antibody (denosumab) that inhibits RANK [102]. These findings underline the potential role of the RANK/RANKL pathway in BC and may lead to new options, for both prevention and therapy, by specifically inhibiting RANK.

### 3.4. Androgens and the Androgen Receptor in TNBC

Although TNBC is characterized by the absence of ER, PR and HER2 receptor expression, 12–55% of TNBCs show androgen receptor (AR) expression on IHC [103]. Gene expression analysis of TNBC identifies a very distinct subtype of TNBC expressing AR, termed the Luminal Androgen Receptor (LAR) subtype, comprising about 9–16% of TNBCs [104,105]. The presence of AR expression among TNBC is generally associated with a better prognosis than TNBC without AR expression (reviewed in [106,107]), although there is some disagreement on this [108].

Testosterone is a strong agonist of AR, and can be converted locally by 5α-reductase, into the much more potent dihydrotestosterone (DHT) inside specific peripheral tissues (reviewed in e.g., [109]). In contrast, androstenedione and dehydroepiandrosterone (DHEA) are very weak agonists of AR, which in the presence of DHT or testosterone may even act as (competitive) antagonists and are therefore mostly considered to be precursors of testosterone [110]. However, the possibility exists that TNBC cells have the ability to convert DHEA into testosterone or DHT intracellularly, and DHEA levels could therefore be important as well [111,112,113,114,115].

Thus, increased androgen levels could negatively influence prognosis of AR+ TNBC. Conversely, blockade of the AR pathway by anti-androgens appears to suppress tumor growth, migration, invasion, and induces apoptosis [104,116,117]. Within the context of this review, the most important aspect for TNBC patients would therefore be how serum androgen levels are affected by menopausal status and hormone replacement therapy. Important in the context of HRT is that despite structural biochemical similarity, 17β-estradiol, and progesterone (both used in HRT) most likely have no meaningful stimulatory effect on the androgen receptor under physiological conditions [110,112,118,119,120].

In general, women have much lower serum testosterone and DHT levels than men, while androstenedione and DHEA levels are about the same (serum level of DHEA in women is 10–20 times that of testosterone). Testosterone and DHT levels further decrease with age, especially when estrogen replacement therapy is given [121,122]. DHEA levels decline gradually with age, but are not significantly affected by either natural or surgical menopause [122]. DHEA-S (a reservoir of DHEA) levels were not affected by HRT in one study, and only mildly reduced in another [121,123]. Finally, HRT also increases serum levels of proteins that bind androgens, reducing bioavailability even further, although DHEA is again not strongly affected [121,122,123]. Summarizing, serum concentrations and/or bioavailability of most important natural androgens decrease gradually with age and sometimes with HRT as well, but not necessarily due to menopause itself.

Lacking a meaningful direct effect on AR, HRT could theoretically have a positive impact on the prognosis of AR positive TNBC, through reduction of free serum androgens (as demonstrated in Figure 1, pathway V). Direct blockade of AR would probably be more effective however, considering the potential for intra-tumor steroidogenesis. Nonetheless, there seems to be no indication of tumor-promoting effects of estrogens or progestogens on AR positive TNBC, which is the main concern when prescribing these drugs in cancer survivors. In fact, when GPER is also expressed by an AR positive TNBC, GPER agonism (such as by E2) may even reduce the growth-stimulating potential of DHT [117]. Considering the incidental finding of AR-activation at physiological progesterone levels [118], caution in its prescription may be warranted for AR+ TNBC patients until further evidence of safety is found.

## 4. Conclusions

Under the dogma that TNBC/HR-negative BC cannot be affected by estrogens and progestogens, many incidental findings in the past decennia showing the contrary may have been written off as random effects of chance or poor methodology (i.e., EBCTG 2005). More recent advances in breast cancer research and molecular techniques have elucidated the possibility that both estrogens and progestogens can circumvent the absence of their respective traditional nuclear receptors in TNBC. Although much work has been done, a lot is still unclear about these alternative pathways. A schematic overview of the reviewed pathways is provided in Figure 1. A more in-depth overview of the effects of estrogens and progestogens on each pathway is provided in Table 1.

From a clinical standpoint, prescription of progestogens and estrogens may or may not be harmful to TNBC patient outcomes, depending on RANK, ERβ, and GPER expression patterns and their interactions with other genes, such as mutant *TP53*. Even in the absence of direct hormone pathways for estrogens at time of diagnosis, changes in receptor expression of TNBC (conversion) or indirect effects (via androgen levels) on for example AR-positive TNBC need to be considered. Ideally, in the future we will be able to map all of these pathways in a similar manner as to how personalized BC recurrence risk assessments are currently used (e.g., Oncotype DX^®^), and be able to provide a personalized recommendation on the use of HRT or the necessity for suppression of ovarian function. In closing, not enough evidence to prevent spontaneous menstrual cycle recovery exists, and at the same time there appears to be too little certainty to allow HRT for postmenopausal symptoms in TNBC patients.

## Figures and Tables

**Table 1:** Schematic overview of direct and indirect effects of estrogen and progesterone on TNBC.

ERβ	Direct Effect	
	Favorable	Unfavorable
	Inhibition by tamoxifen in vitro [124] and clinical studies [61,62,63,64] Preclinical studies: Inhibition of epithelial-to-mesenchymal-transition (EMT) [37] Inhibition of invasion and migration [38,39] Inhibition of proliferation in ERβ transfected cells [32,40] Inhibition of proliferation when ERβ is upregulated [41] Interference with mutant TP53 [42,46], often present in clinical TNBC [47] and TNBC cell lines [48,49] Blockade of cell cycle [43] Activation of cystatins that block TGFβ pathway [44,45]	Clinically unfavorable outcome [27,50,58,60,61] ERβ2 associated with worse overall survival in TNBC patients [54] Preclinical studies: Activation of ERβ by selective ERβ agonists [125,126], stimulates proliferation, invasion and migration of TNBC cell lines expressing ERβ (both mRNA and protein) [50] Stimulation of proliferation in general [51] Promoting cell survival via fatty acid uptake (FATP1) [52] ERβ-knockout reduces tumor-aggressiveness [50,53] Interaction with other proteins, such as pAKT [59,127] Isoforms (splice variants) of ERβ (five known currently, including wild type ERβ1) such as ERβ2 or ERβ5 may negatively affect prognosis [35,57,128,129,130] ERβ2 and ERβ5 expression, but not ERβ1 expression, is found in commonly used TNBC cell lines [54] ERβ2 and ERβ5 expression associated with increased proliferation, migration and invasiveness of TNBC cell lines. ERβ1 expression suppresses proliferation, migration and invasiveness of TNBC cell lines [54]
**ERβ**	**Indirect Effect**	
	**Favorable**	**Unfavorable**
	Interference with growth-stimulating potential of GPER [79] Interference with growth-stimulating and metastatic potential of AR [131,132]	
**RANK**	**Indirect Effect**	
	**Favorable**	**Unfavorable**
		Progesterone stimulates RANK-ligand production in neighboring cells, causing indirect stimulation of the RANK-expressing cancer cell [81,82] Effect is increased proliferation, metastatic potential and therapy resistance, resulting in decreased survival [82,85,86,87,88] RANK/RANKL may play a key role in carcinogenesis in BRCA1 mutation carriers [100,101,102], and RANK-inhibition may a be an effective prevention and treatment option [102]
**AR**	**Direct Effect**	
	**Favorable**	**Unfavorable**
	No meaningful direct effect of estrogen, and most likely of progesterone, on AR. [110,112,118,119,120]	
**AR**	**Indirect Effect**	
	**Favorable**	**Unfavorable**
	Possible benefit through reduction of free serum androgens [121,122,123] (as stimulation of AR is likely detrimental [104,116,117]), but is subject to potential for intracellular DHEA conversion by TNBC [111,112,113,114,115]	
**GPER**	**Direct Effect**	
	**Favorable**	**Unfavorable**
		Most likely unfavorable, but may depend on expression of other proteins, such as ERβ. Clinical data [80] Preclinical data [74,75,76]
**GPER**	**Indirect Effect**	
	**Favorable**	**Unfavorable**
	Stimulation of GPER suppresses, rather than promotes migration when ERβ is also expressed [79]	
**ERα and PR**	In the event of receptor conversion, possible stimulation of growth and metastatic potential [12]	

## Data Availability

Data sharing is not applicable to this article, as no new data were created or analyzed.
